# Supplementary material for: Multi‐dataset identification of innovative feature genes and molecular mechanisms in keratoconus
Source: J Cell Mol Med. 2024 Sep 19;28(18):e70079. doi: 10.1111/jcmm.70079 (PMC11412914; doi:10.1111/jcmm.70079)
Supplement: Supplementary file 2 — Material S2. [file JCMM-28-e70079-s002.zip › 7.GSEA analysis/3.DBNDD1/KEGG_RIBOSOME.html]

Details for gene set KEGG\_RIBOSOME[GSEA]

|  || Dataset | DBNDD1.group.cls#h\_versus\_l |
| Phenotype | group.cls#h\_versus\_l |
| Upregulated in class | h |
| GeneSet | KEGG\_RIBOSOME |
| Enrichment Score (ES) | 0.29746357 |
| Normalized Enrichment Score (NES) | 1.1115876 |
| Nominal p-value | 0.31150794 |
| FDR q-value | 1.0 |
| FWER p-Value | 0.996 |
Table: GSEA Results Summary

  

Fig 1: Enrichment plot: KEGG\_RIBOSOME      
 Profile of the Running ES Score & Positions of GeneSet Members on the Rank Ordered List

  

| SYMBOL | TITLE | RANK IN GENE LIST | RANK METRIC SCORE | RUNNING ES | CORE ENRICHMENT || 1 | RPS4Y1 | na | 1136 | 0.369 | 0.1816 | Yes |
| 2 | RPL27A | na | 5067 | 0.178 | 0.1717 | Yes |
| 3 | RPL3L | na | 7625 | 0.111 | 0.1627 | Yes |
| 4 | RPL32 | na | 9859 | 0.073 | 0.1408 | Yes |
| 5 | RPS20 | na | 12079 | 0.046 | 0.1037 | Yes |
| 6 | RPS9 | na | 12476 | 0.043 | 0.1171 | Yes |
| 7 | RPS2 | na | 12877 | 0.039 | 0.1284 | Yes |
| 8 | RPS3 | na | 12903 | 0.039 | 0.1503 | Yes |
| 9 | RPS23 | na | 13479 | 0.034 | 0.1537 | Yes |
| 10 | RPL22 | na | 14713 | 0.025 | 0.1326 | Yes |
| 11 | RPL39 | na | 14879 | 0.024 | 0.1416 | Yes |
| 12 | RPL36 | na | 15141 | 0.022 | 0.1468 | Yes |
| 13 | RPL13 | na | 15570 | 0.019 | 0.1453 | Yes |
| 14 | MRPL13 | na | 15619 | 0.018 | 0.1545 | Yes |
| 15 | RPS15A | na | 15898 | 0.017 | 0.1562 | Yes |
| 16 | RPL18A | na | 15915 | 0.016 | 0.1653 | Yes |
| 17 | RPL10L | na | 16040 | 0.016 | 0.1708 | Yes |
| 18 | RPL34 | na | 16142 | 0.015 | 0.1765 | Yes |
| 19 | RPS17 | na | 16272 | 0.014 | 0.1809 | Yes |
| 20 | RPL6 | na | 16330 | 0.014 | 0.1872 | Yes |
| 21 | RPS29 | na | 16352 | 0.013 | 0.1945 | Yes |
| 22 | RPL4 | na | 16385 | 0.013 | 0.2013 | Yes |
| 23 | RPS7 | na | 16636 | 0.012 | 0.2009 | Yes |
| 24 | RPL36AL | na | 16640 | 0.012 | 0.2076 | Yes |
| 25 | RPL29 | na | 16665 | 0.011 | 0.2136 | Yes |
| 26 | RPL36A | na | 16721 | 0.011 | 0.2185 | Yes |
| 27 | RPL23 | na | 16748 | 0.011 | 0.2241 | Yes |
| 28 | RPLP0 | na | 16760 | 0.011 | 0.2302 | Yes |
| 29 | RPLP2 | na | 16765 | 0.011 | 0.2364 | Yes |
| 30 | RPL15 | na | 16846 | 0.010 | 0.2401 | Yes |
| 31 | RPL8 | na | 17005 | 0.009 | 0.2410 | Yes |
| 32 | RPL35 | na | 17061 | 0.009 | 0.2446 | Yes |
| 33 | RPL11 | na | 17106 | 0.009 | 0.2484 | Yes |
| 34 | RPS28 | na | 17109 | 0.009 | 0.2534 | Yes |
| 35 | RPL30 | na | 17119 | 0.009 | 0.2582 | Yes |
| 36 | RPL41 | na | 17205 | 0.008 | 0.2604 | Yes |
| 37 | RPL26 | na | 17281 | 0.008 | 0.2626 | Yes |
| 38 | RPL18 | na | 17359 | 0.007 | 0.2646 | Yes |
| 39 | FAU | na | 17393 | 0.007 | 0.2677 | Yes |
| 40 | RPS10 | na | 17427 | 0.007 | 0.2706 | Yes |
| 41 | RPL31 | na | 17447 | 0.007 | 0.2740 | Yes |
| 42 | RPL13A | na | 17454 | 0.007 | 0.2776 | Yes |
| 43 | RPL38 | na | 17600 | 0.006 | 0.2767 | Yes |
| 44 | RPL5 | na | 17604 | 0.006 | 0.2798 | Yes |
| 45 | RPS19 | na | 17609 | 0.006 | 0.2830 | Yes |
| 46 | RPL9 | na | 17657 | 0.005 | 0.2847 | Yes |
| 47 | RPL27 | na | 17665 | 0.005 | 0.2875 | Yes |
| 48 | RPL12 | na | 17674 | 0.005 | 0.2903 | Yes |
| 49 | RPS13 | na | 17745 | 0.005 | 0.2910 | Yes |
| 50 | RPS18 | na | 17748 | 0.005 | 0.2936 | Yes |
| 51 | RPL14 | na | 17903 | 0.003 | 0.2912 | Yes |
| 52 | RPS8 | na | 17924 | 0.003 | 0.2925 | Yes |
| 53 | RPL23A | na | 18006 | 0.003 | 0.2917 | Yes |
| 54 | RPS16 | na | 18089 | 0.002 | 0.2905 | Yes |
| 55 | RPL3 | na | 18148 | 0.002 | 0.2898 | Yes |
| 56 | RPL37A | na | 18156 | 0.002 | 0.2905 | Yes |
| 57 | RPL26L1 | na | 18174 | 0.002 | 0.2909 | Yes |
| 58 | RPS27L | na | 18240 | 0.001 | 0.2897 | Yes |
| 59 | RPS12 | na | 18257 | 0.001 | 0.2898 | Yes |
| 60 | RPL17 | na | 18411 | -0.000 | 0.2855 | Yes |
| 61 | RPS25 | na | 18422 | -0.000 | 0.2854 | Yes |
| 62 | RPS15 | na | 18491 | -0.001 | 0.2838 | Yes |
| 63 | RPL7A | na | 18561 | -0.001 | 0.2826 | Yes |
| 64 | RPS11 | na | 18603 | -0.002 | 0.2823 | Yes |
| 65 | RPL37 | na | 18641 | -0.002 | 0.2823 | Yes |
| 66 | RPL7 | na | 18655 | -0.002 | 0.2831 | Yes |
| 67 | RPL24 | na | 18719 | -0.002 | 0.2827 | Yes |
| 68 | RPL22L1 | na | 18749 | -0.003 | 0.2834 | Yes |
| 69 | RPS3A | na | 18778 | -0.003 | 0.2842 | Yes |
| 70 | UBA52 | na | 18783 | -0.003 | 0.2858 | Yes |
| 71 | RPS5 | na | 18817 | -0.003 | 0.2866 | Yes |
| 72 | RPL19 | na | 18822 | -0.003 | 0.2883 | Yes |
| 73 | RPL21 | na | 18991 | -0.004 | 0.2860 | Yes |
| 74 | RPS27 | na | 19026 | -0.005 | 0.2878 | Yes |
| 75 | RPS27A | na | 19317 | -0.007 | 0.2835 | Yes |
| 76 | RSL24D1 | na | 19382 | -0.008 | 0.2861 | Yes |
| 77 | RPL10A | na | 19522 | -0.009 | 0.2871 | Yes |
| 78 | RPL35A | na | 19851 | -0.012 | 0.2844 | Yes |
| 79 | RPLP1 | na | 19864 | -0.012 | 0.2909 | Yes |
| 80 | RPS26 | na | 19993 | -0.013 | 0.2948 | Yes |
| 81 | RPS6 | na | 20247 | -0.015 | 0.2963 | Yes |
| 82 | RPS4X | na | 20568 | -0.018 | 0.2975 | Yes |
| 83 | RPS21 | na | 22023 | -0.032 | 0.2743 | No |
| 84 | RPS24 | na | 25470 | -0.078 | 0.2201 | No |
| 85 | RPL28 | na | 25637 | -0.081 | 0.2624 | No |
Table: GSEA details [plain text format]

  

Fig 2: KEGG\_RIBOSOME      
 Blue-Pink O' Gram in the Space of the Analyzed GeneSet

  

Fig 3: KEGG\_RIBOSOME: Random ES distribution      
 Gene set null distribution of ES for **KEGG\_RIBOSOME**
